# Systems Biology Approaches to Decipher the Underlying Molecular Mechanisms of Glioblastoma Multiforme

**DOI:** 10.3390/ijms222413213

**Published:** 2021-12-08

**Authors:** Ali Kaynar, Ozlem Altay, Xiangyu Li, Cheng Zhang, Hasan Turkez, Mathias Uhlén, Saeed Shoaie, Adil Mardinoglu

**Affiliations:** 1 Centre for Host-Microbiome Interactions, Faculty of Dentistry, Oral and Craniofacial Sciences, King’s College London, London SE1 9RT, UK; ali.kaynar@kcl.ac.uk (A.K.); saeed.shoaie@scilifelab.se (S.S.); 2 Science for Life Laboratory, Royal Institute of Technology (KTH), SE-10691 Stockholm, Sweden; oaltay@kth.se (O.A.); xiangyu.li@scilifelab.se (X.L.); cheng.zhang@scilifelab.se (C.Z.); mathias.uhlen@scilifelab.se (M.U.); 3 Medical Biology Department, Faculty of Medicine, Atatürk University, Erzurum TR-25240, Turkey; hasanturkez@yahoo.com

**Keywords:** glioblastoma, genome-scale metabolic models, multi-omics data, systems biology

## Abstract

Glioblastoma multiforme (GBM) is one of the most malignant central nervous system tumors, showing a poor prognosis and low survival rate. Therefore, deciphering the underlying molecular mechanisms involved in the progression of the GBM and identifying the key driver genes responsible for the disease progression is crucial for discovering potential diagnostic markers and therapeutic targets. In this context, access to various biological data, development of new methodologies, and generation of biological networks for the integration of multi-omics data are necessary for gaining insights into the appearance and progression of GBM. Systems biology approaches have become indispensable in analyzing heterogeneous high-throughput omics data, extracting essential information, and generating new hypotheses from biomedical data. This review provides current knowledge regarding GBM and discusses the multi-omics data and recent systems analysis in GBM to identify key biological functions and genes. This knowledge can be used to develop efficient diagnostic and treatment strategies and can also be used to achieve personalized medicine for GBM.

## 1. Introduction

Glioblastoma (GBM) is an aggressive and malignant cancer of the central nervous system (CNS) [1,2], with a high incidence rate (3.23 per 100,000) [3]. Based on the increasing evidence that early cancer diagnosis is life-saving [4], discovering biomarkers that could be detected before the clinical presentation of cancer symptoms might improve survival. Currently, the standard treatment approaches for GBM are surgical, radiotherapy, and chemotherapy [5]. However, recurrence is one of the leading causes of the low survival rate of GBM [6,7].

The increment of biological data obtained with the enhanced technology and emergence of multi-omics approaches could lead to a reinterpretation of the molecular mechanisms of GBM and enable the development of treatment approaches. The systems biology methodologies provide a systems-level assessment of molecular events to find drug targets for therapeutic purposes. Data obtained by high-throughput screening and computational biology methods open up avenues of repurposing approved chemical agents as drugs with tolerable side effects for cancer patients [8]. Moreover, innovative treatment approaches, such as drug repositioning, might help in finding rapid solutions in the fight against GBM [8].

## 2. Biological Basis of Glioblastoma

### 2.1. Epidemiology and Classification

Although the global incidence of CNS cancers (1.7%) is less than the other types of cancer, GBM is one of the cancer types with the highest mortality rate (median survival 8 months) [3,9]. Today, GBM accounts for 14.5% of all cancer types in CNS and 48.6% of malignant CNS tumors [3,9]. According to the WHO classification of CNS tumors (5th edition, 2021), glioblastomas have been divided into primary and secondary. Primary glioblastoma is defined as diffuse astrocytic tumors in adults that must be IDH-wildtype, and secondary glioblastoma equates to astrocytoma, IDH mutant, WHO CNS grade-4 [10,11]. In addition, three variants are recognized, which are giant cell glioblastoma, gliosarcoma, and epithelioid glioblastoma. The genetic criteria for a diagnosis of IDH-wildtype glioblastoma are stated as TERT promoter mutation or EGFR gene amplification or +7/−10 chromosome copy number changes [11]. Of note, in younger age groups, the diagnosis should be based on the different types of diffuse pediatric-type gliomas [11].

### 2.2. Pathogenesis: Molecular Features and Genomic Alteration

Molecular studies have revealed major genetically based events in the development of GBM: (1) Retinoblastoma tumor suppressor and p53 deactivation pathways; (2) phosphatidylinositol-3-OH kinase (PI3K) pathway activation; (3) growth factor signaling defects depending on receptor tyrosine kinase (RTK) activation [12,13]. Since the WHO-based classification is a histological classification, it may not be sufficient to distinguish between molecular differences and genetic alterations. Therefore, molecular features of GBM could help improve the classification. For example, classical, mesenchymal, and proneural subtypes are mainly classified according to the molecular subtyping [14]. Differences in gene expression profiles of *PDGFRA-IDH1*, *EGFR*, and *NF1* could be used to determine the proneural, classical, and mesenchymal types, respectively [13]. Moreover, genomic screening may identify more profound and more extensive oncogenic alterations. According to The Cancer Genome Atlas (TCGA) study, the highly deregulated signaling pathways in GBM are RTK/PI3K signaling pathways, P53, RB, RTK/RAS, PI3K class 1/AKT, and PI3K class 2 signaling pathways. These pathways are involved in cell migration, cell survival, cell cycle, DNA repair, and apoptosis mechanisms, known as typical cancer cell hallmarks [15].

In the last few years, CRISPR technology has expeditiously revolutionized the genetic research, by enabling the investigation of the molecular mechanisms in tumorigenesis and revealing the targets for drug development. Forward genetic screens using CRISPR-Cas9 systems have been performed to identify the driver genes for cell survival in solid tumors, including GBM. For instance, MacLeod et al. identified the members of SOX transcription factor family (SOCS3, USP8, and DOT1L) and protein ufmylation are the regulators of the growth of glioblastoma stem cells [16]. Similarly, Prolo et al. showed the role of MAP4K4 in the glioblastoma invasion using CRISPR-Cas9 loss of function screens [17].

### 2.3. Heterogeneity

Histological studies reveal that GBM is a highly heterogeneous tumor regarding cell types, cell sizes, cell density, mitosis number, vascularization, and necrosis distribution. The integrated multi-dimensional genome sequence, transcriptomic, and epigenomic analysis helped discover and categorize oncogenic somatic alterations, in addition to the cancer candidate genes shown in previous studies (e.g., *TP53*, *MDM2*, *MDM4*, *PTEN*, *NF1*, *EGFR*, *CDKN2A*, *RB1*, *CDK4*, *PIK3R*, *PIK3CA*, *PIK3R1*, and *IRS1*) [18,19,20]. Today, only a few large-scale genomic and epigenetic studies have enabled the discovery of new candidate genes and helped in understanding the heterogeneity [12]. As an example, non-GBM-related *IDH1* mutation and chromosomal abnormalities, such as somatic mutations or copy number changes, are shown to contribute to heterogeneity [19]. Heterogeneity could be distinguished based on the gene expression profile, as in the distinction of GBM subtypes, such as proneural, neural, classical, and mesenchymal. For example, the proneural subtype with intact *PTEN* and normal *EGFR* expression could be characterized by the expression of genes associated with normal brain tissue and neurogenesis [20]. In parallel, DNA methylations, especially hypermethylation of CpG islands, is one of the hallmarks of human cancers. Hypermethylation of the CpG island in the promoter region often causes transcriptional silencing of the associated gene by blocking the required transcription factors’ binding to transcription initiation sites. DNA methylations have been found in promoter-associated CpG island hypermethylation in human GBM and other glioma subtypes [18]. The miRNA alteration is another critical factor in post-translational modifications and plays a crucial role in modifying genes involved in carcinogenesis, cancer invasion, and escaping from apoptosis mechanisms [18]. Clinically, according to the miRNA expression profile, GBM is divided into five subtypes: Astrocytic, neural, oligoneural, radial glial, and neuromesenchymal [21].

### 2.4. Tumor Microenvironment

Solid cancers change their microenvironment by utilizing intercellular matrices. In addition, GBM shows the same behavior. However, it usually does not spread to localized niches outside CNS. Instead, it is in the region where it originated and is protected from the immune system behind the BBB. However, it has also been reported that recurrent GBM might occur at a location farther from the region where it has first appeared. Therefore, the therapy resistance due to dynamics and a poorly accessible microenvironment pose a great challenge to treat GBM [22].

The GBM microenvironment comprises proliferative malignant astrocytoma, cancer stem cells, pericytes, stromal, vascular endothelial, and immune cells. These cells create different niches, including angiogenic tumor niche, invasive tumor niche, and hypoxic tumor niche. Moreover, in these niches, different tumor cell types and non-cancerous cells (e.g., microglia, dendritic cells, lymphocytes, macrophages) change the form dynamically by interacting within the extracellular matrix framework. Although these niches provide features, such as metabolic abnormality, cell growth, invasion, and glioma stem cell production, the typical feature is the vasculature in the niche environment. For example, tumor cells move toward abnormal angiogenic vasculature in niches. On the other hand, tumor cells migrate into the brain parenchyma using ordinary blood vessels in invasive niches. Finally, in the hypoxic niche, a necrotic area surrounded by hypoxic tumor cells is formed [23].

### 2.5. Crosstalk between Tumor Cells and Their Microenvironment

Studies have shown that microglial or astrocytes stimulate the growth of GBM [24]. There is also evidence that tumor cells communicate with neurons through synaptic connections [25]. The interaction of tumor cells with microglia and astrocytes are orchestrated by the changes in the extracellular matrix and cytoskeleton. These interactions are established by non-coding RNA, cytokines, chemokines, neurotrophic factors, morphogenic factors, ABC transporters, extracellular vesicles, and metabolic factors [25]. Some metabolites protect tumor cells from the immune system by forming an immunosuppressive microenvironment [26].

During the progression of GBM, the BBB allows immune cells to enter the blood circulation and causes neuroinflammation, which induces glial activation and chemoattraction. Marker studies have shown that microglial cells have a pro-tumor phenotype [24]. For instance, microglial cells produce plenty of pro-inflammatory substances, such as nitric oxide (NO), tumor necrosis factor-alpha (TNF-α), and other interleukins (ILs), that stimulate astrocyte activation and cause progressive BBB degradation, which can in turn cause irreversible BBB degradation. Furthermore, it has been shown that glial cells in the tumor microenvironment stimulate tumor proliferation by expressing arginase-1 (ARG-1). In addition, tumor cells induce astrocytes to be more reactive by interacting with astrocytes/microglial cells through the extracellular vesicles [24].

### 2.6. Diagnosis and Current Therapy Approach

**Figure 1:**
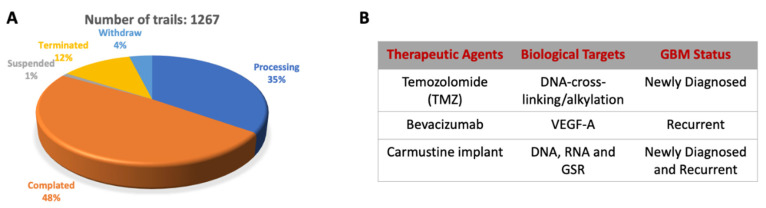
Clinical trials status and current therapeutic agents for GBM. (**A**) The total number of clinical trials for GBM is 1267. (**B**) Approved therapeutic agents used for the GBM treatment.

The current GBM therapy includes radiotherapy and chemotherapy following surgery (Figure 1). However, due to the molecular heterogeneity of GBM, tumor recurrence is highly possible. Therefore, some genetic markers (e.g., *IDH1/IDH2* mutations, *MGMT* promoter methylation, *EGFR* mutation/amplification, *TP53* mutation, *PTEN* mutation) and medical imaging have been used as a prognostic prediction in clinical trials (Figure 1) [27].

Following the development of sequencing technologies and advances in multi-omics analyses, personalized therapeutic approaches have become possible. Therefore, new GBM treatment approaches, such as antibody-based drugs, vaccines, growth factor receptors targeted molecular inhibitors, inhibitors blocking immune checkpoints, immune system redirection therapies, and tumor-targeted oncolytic viruses, are some approaches under development [28].

## 3. Systems Biology Approaches to GBM

Several biological data sources and disease models have been established to help researchers characterize the disease, develop treatment approaches, and conduct drug studies to tackle GBM. This knowledge could be derived from in vitro, in vivo, and in silico studies. However, the GBM tumor extracted by surgical treatment and post-mortem tissue is the primary source of biological data. Moreover, to produce biological data, GBM models made by both animal and cell lines could be developed by silencing tumor suppressors, such as *P53*, *NF1*, *PTEN*, *RB/p107/p130* or the activation of oncogenes, such as *EGFR*, *HRasV12*, *KRasG12D*, *PDGF*, and virus intervention [29,30]. Scientists could multi-dimensionally investigate GBM metabolism through multi-omics approaches and generate a wide variety of biological data through studies with cell lines, model organisms, and post-mortem tissues. In addition, metabolite measurements from the cerebrospinal fluid and blood facilitate the range of information and biomarker studies. With the enhancement in the high-throughput technologies, the multi-omics approaches can cover the central dogma and its beyond.

Systems biology is a holistic approach focusing on the relationship between biological components rather than individual molecules to decipher biological systems’ complexity [8,31]. Systems biology approaches could comprehensively evaluate the interpretation of multi-omics data derived from wet-lab experiments. The success of systems biology approaches, which aim to perform realistic predictions regarding biological conditions, is closely dependent on the quality of the measured data and the computational methods [32]. Human, technical, and environmental factors may cause bias in human tissue samples and biological data [33]. Published datasets that passed the quality control are considered safe for further analyses (Table 1).

With the system biology approaches, critical gains, such as classification, finding biomarkers, and drug repurposing have been achieved [8]. Therefore, the analysis of GBM multi-omics data to unveil the underlying molecular mechanisms of GBM and possible treatment approaches is promising.

The system biology approach has been applied to studies tackling complex diseases, such as hepatocellular carcinoma, liver disease, and GBM [8,34,40,41], and promising results have been obtained.

### 3.1. Omics Data

High-throughput methods are available to measure the biological conditions and alterations resulting from the transmission of genetic information through the whole biological network. DNA is the source of biological information that forms the genomics layer. DNA level alteration, such as mutation and copy number variation, directly affect cellular functions. Genomic and epigenomic profiles involve the mechanisms regulating gene transcription events, including transcription factors and post-translational modifications, which affect the structure and quantity of mRNA levels that form the transcriptomic layer. Proteomics measure the protein levels in a biological sample, whereas metabolomics determine the cellular metabolic activity due to enzyme activities. Measurement of the flow rate of metabolites in intracellular reactions is defined by fluxomics. Interactomics are also crucial to study in protein-protein interaction networks (PPINs) and regulatory networks (RNs). Although each omics layer is valuable separately, transcriptomics and proteomics are used by a majority in multi-omics integration studies within the scope of system biology [42].

#### 3.1.1. Transcriptomics

Transcriptomics related technologies empower scientists for the measurement of the RNA profile of an organism. Transcriptomics point out an instantaneous structure of the entire mRNA transcripts expressed in a cell. Two techniques are mainly used today. The first is microarray technology, a precursor to the transcriptome profiling technique that measures mRNA levels using predetermined gene-specific sequences printed on the array chip. The second is RNA-Seq, the state-of-the-art RNA sequencing technique that uses high-throughput sequencing to capture all of the RNA content [43].

- Microarray-based transcriptomics of GBM

In the first microarray studies, a limited number of genes were identified in the microarray library. Atlas^®^ Human cDNA Expression Array was used in the first microarray study for GBM. Only 597 genes, including 588 known and nine housekeeping genes, have cDNAs on the membrane to determine the expression level [44]. This study compared the gene expression profile of GBM and normal brain tissue, which is the first comparative genomics. The first differentially expressed gene (DEG) analysis determined that the *CXCR-4* gene has high expression in GBM. The relationship between the overexpression of the *CXCR-4* and neurite outgrowth and cellular differentiation have been identified [44]. In another study, the gene expression profile was measured for 5760 individual targeted genes and found that 117 genes were up- and 111 genes were downregulated. Specifically, overexpression of the *SPARC*, *IGFBP2*, and *VEGF* was observed for the GBM profile [45].

Advances in technology and the creation of more comprehensive microarray chips covered more genes and allowed for extensively profiling changes in metabolism. The first microarray data published in the literature, GSE1923, was stored in the Gene Expression Omnibus (GEO) database. In this study, 594 of 12,625 genes were significantly altered and covered more genes than previous studies. This study identified a complex gene expression profile resulting from PDGF-associated signaling in U87 MG glioblastoma cells [46]. Although it was advanced technology in the early days, the microarray has significant disadvantages. First, it needs a reference genome and depends on probes. Second, it excludes uncharacterized genes. The stabilization of chips is another critical issue that can cause inaccurate gene expression profiling. Nevertheless, it has made a significant contribution to science by measuring numerous gene expressions simultaneously in its time by establishing pioneer system-based approaches.

b. Next-Generation Sequencing based transcriptomics

With the advancement in sequencing technologies, which has increased the importance of the big data concept in biology, a plethora of biological data have been produced that can be used to understand complex disease phenotypes. The most outstanding contribution to science is its ability to generate massive volumes of data, as well as its ability to provide fast, cheap, and accurate genome information. NGS technology can be classified as second and third generation. While second-generation sequencing methods require the amplified sequencing libraries arrangement before sequencing the amplified DNA clones, third-generation sequencing could be performed without the arrangement of amplification libraries. In 2000, the first NGS technology was developed by massively parallel signature sequencing (MPSS, Lynx Therapeutics, Hayward, CA, USA). In the following years, the appearance of multiple companies that develop NGS machines contributed to a price reduction and offered alternatives. The rapid advancement in NGS technology has contributed to the simultaneous development of systems biology approaches. NGS has become widely used for whole-genome, whole-exome, methylation, and RNA sequencing. The RNA sequencing could be used to determine the whole transcriptional activities, both coding and non-coding RNA, as well as the targeted RNA transcripts in a biological sample. It provides a more accurate and precise measurement of gene expression than microarray analysis. In addition, RNA-Seq does not require a reference genome as in the microarray. Therefore, RNA-Seq is an attractive method for discovering new genes. RNA sequence is based on annealing and synthesis rather than hybridization. Consequently, it is not affected by error sources, such as background noise and cross-hybridization, and it is advantageous in detecting the profile of low-expression transcripts.

The first RNA-Seq data for GBM analysis was published in 2011 and was performed for only two samples: Peripheral brain and tumor brain tissue samples. An RNA-Seq analysis pipeline, named RNASEQR, was developed, and the results were compared with genome and transcriptome references. As a result, numerous new and previously unmapped gene regions were identified [47]. Another pioneer RNA-Seq study showed that *Atf3*, *Cbx7*, and a few other candidates are tumor suppressor genes in human GBM. In addition, potential oncogenes, such as *Ccnd2* and *Klf6*, have been revealed [48].

#### 3.1.2. Proteomics Data

Proteomics datasets offer an opportunity to examine the biology and disease mechanism of GBM on the genotype-phenotype axis by analyzing snapshots of metabolic pathways, signaling pathways, and complex interaction networks [49]. In the flow of information through the central dogma, the protein information after transcription is critical for the cell to reflect essential functions, such as enzymatic activity, cell structure, and signal transmission.

The first liquid chromatography-mass spectrometry (LC-MS) study conducted for GBM was a protein-protein interaction research focusing on mitochondrial proteins. Nine hundred and two proteins were measured simultaneously and explained the changes in the protein levels due to oxidative stress and abnormal energy metabolism [50]. Proteomics data have the potential to classify the GBM subtypes. For example, the SERPINE1 protein was distinguished by overexpression in the mesenchymal subtype [51,52]. Although the transcriptomics data contain much more gene information, the mRNA level may not strongly reflect the protein level due to post-translational modifications that may affect stability. Cellular activities mainly depend on functional proteins [51]. Therefore, the metabolic network controlled by enzymatic reactions might more realistically reflect cellular information through protein information.

### 3.2. Network Analysis of GBM

**Figure 2:**
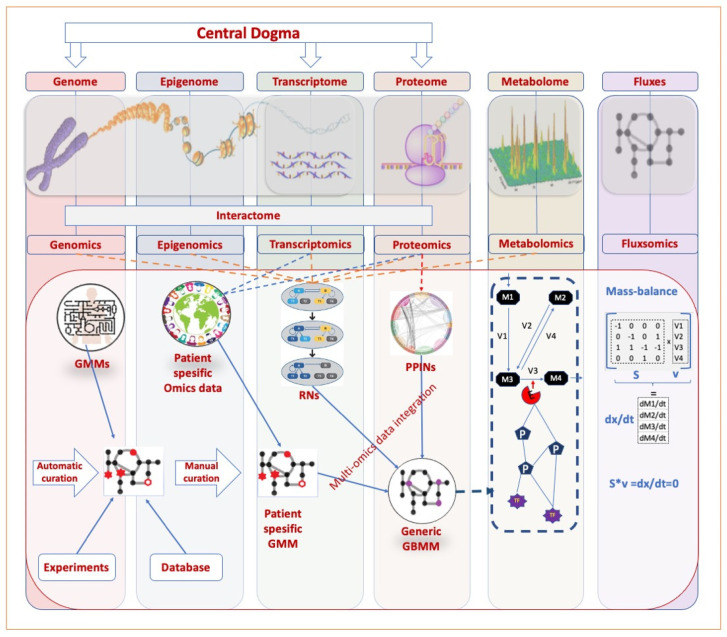
Multi−omics integration perspective. The central dogma describes the flow of genetic information within a biological system. In multi-omics approaches, different ohmic layers can be used separately or together for various purposes. The glioblastoma-specific metabolic model (GBMM) is developed using generic metabolic models (GMMs), experimental results, databases, and patient-specific omics data. Regulatory networks (RNs) are clusters of macromolecules belonging to different omics layers that interact to control the expression level of various genes. Protein-protein interaction networks (PPINs), formed by the interaction of two or more proteins, contain the information of the subnetworks associated with the disease. Using RNs, PPINs, and GBMM together, the effect of different omics layers on the metabolic profile can be estimated. M represents the metabolite (M1, M2, M3, M4); E represents the enzyme; P represents protein. TF: Transcription factor; S: Stochiometric matrix; V: Flux rate (V1 V2, V3, V4). The navy dashed-lined quadrangular represents a cell boundary.

Advances in omics technologies have enhanced the evaluation of cancer metabolism by developing a comparative analysis of healthy brain and cancer (Table 1). As a top-down approach, omics technologies provide information regarding complex biological systems at different levels. However, the interaction between the omics data creates an increased solution space on the complexity of biological systems. Therefore, holistic approaches are required to evaluate the functions of biological systems (Figure 2).

### 3.3. Genome-Scale Metabolic Network Modeling

Genome-scale metabolic models (GEMs) are the mathematical representations of biochemical reactions based on stoichiometry that reflect the metabolic activity within a cell and decipher the information that regulates these reactions [41]. GEMs use the information flow in the central dogma to simulate the reflection of the genotypes to the phenotypes in the cell and are continuously updated with new information. System biology-based studies using GEMs and interactome information (RNs, PPINs, co-expression networks) are available in the literature [6,35,40,41,53,54].

The history of metabolic modeling dates back to the 1990s, aiming to model an entire cell. A low number of metabolites and enzymes was used in the first metabolic models as enzyme kinetics studies [55,56]. On the other hand, GEMS are very effective for flux estimation in small systems by the flux balance analysis (FBA) in the steady-state of the cell [57]. The basic principle is based on the mass balance equation of a metabolite consumed or formed depending on reaction rates. While ordinary differential equations (ODEs) are used in kinetic modeling, FBA is based on the linear equation. Therefore, the techniques developed, such as unstable state FBA (uFBA) could bring flexibility to the steady-state assumption in genome-scale models regarding dynamic cellular states caused by changes in intracellular flux distribution [58]. However, with the increase of multi-omics data, genome-scale metabolic modeling methods have been widely improved. Moreover, some benchmarking studies for methods that create GEMs have been carried out to understand the appropriate method that is suitable for the purpose [35,59].

GEMs comprise acknowledged metabolic reactions of a living system, describing them as a functional reflection of cell-specific metabolism. The procedure for reconstructing a GEM has been systematically explained in the literature [60]. A draft reconstruction is completed by incorporating reactions, enzymes, and gene information from databases such as KEGG, BioCyc, BRENDA, and Human Protein Atlas. Gene-protein-rule (GPR), which includes gene information that controls each reaction, is included in GEMs as in the literature, allowing multi-omics data to be integrated into the model. Various omics data integration strategies may explain tissue-specific and condition-specific intracellular metabolic flux distributions with high resolution. Intracellular metabolic reactions produce basic biochemical building blocks and energy to maintain the basic cellular activities. Based on patient-specific data, personalized GEMs could be generated thanks to the integration of multi-omics data guided by GPR, such as transcriptomics, proteomics, and metabolomics, for personalized medicine. Given the environmental factors on the omics levels, determining the changes to disruptions in intracellular metabolic fluxes and metabolic responses is essential, in order to understand the basic mechanisms of the cell. GEMs powered by omics data are valuable for evaluating the biological system, as they provide the ability to analyze the genotype-phenotype relationship.

In the literature, human generic metabolic models are available. These models contain a wide range of gene, reaction, and metabolite information. These models could be transformed into disease-specific metabolic models with some methods (Table 2). The GBM-specific GEM iMS570g model [54] was developed based on its previous version, the iMS570 model [61], which is based on a comprehensive brain model. The GBM-specific biomass reaction was added to the iMS570g model, and some updates were made by reviewing the literature. Researchers used GIMME and MADE algorithms to map the microarray data to the iMS570g model. This study simulated glycolytic TCA cycle fluxes and the Warburg effect which is followed in the literature in terms of GBM metabolism.

Another GBM-specific metabolic modeling study created GBM generic models to evaluate GBM mechanisms [41]. This study developed a patient-specific GBM metabolic model by integrating patient-specific transcriptome data into the metabolic model using the tINIT algorithm based on the HMR2 metabolic model. As a result of the gene-essentiality analysis, *SOAT1, PGS1*, *CMPK2*, *CRLS1*, and *SLC22A5* targets were estimated as potential drug targets. One of them, *SOAT1*, was proposed as a therapeutic target for GBM [41].

### 3.4. GBM Related Systems Biology Studies

Since GBM is a highly fatal, recurring, and invasive cancer, a limited number of developed drugs are currently in use for its treatment. Another crucial point in combating GBM is the availability of biomarkers. With the emergence of omics data due to the high technology and state-of-the-art approaches, such as system biology, there have been improvements in the diagnosis, treatment, molecular characterization, and determination of subtypes of GBM. In the TCGA project, a comprehensive genomic characterization study for GBM, some significant mutations, and somatic genome alterations were detected, including *TP53*, *EGFR*, *PTEN*, *NF1*, *PIK3CA PIK3R1*, *RB1*, *SPTA1*, *ATRX*, *IDH1*, *KEL*, *PDGFRA,* and *GABRA6*. Moreover, previously uncovered deletions in *PARK2* and *NF1*, and amplifications in AKT3 were detected. Furthermore, the roles of *ERBB2*, *NF1*, and *TP53* genes have been unveiled comprehensively. This genomic screening revealed a link between the hypermutator phenotype in GBM treated with Temozolomide in the context of MGMT-promoter methylation. Another remarkable piece of information is that all of the 38 mutations in the *TP53* gene were detected in the DNA binding domain (The Cancer Genome Atlas Research Network, 2008). Analysis of genome-wide methylation data with systems-based approaches revealed biologically distinct GBM subtypes [18]. The study explained that the DNA methylation pattern of the MGMT gene promoter occurs in 48.5% of GBM patients. In addition, the methylation profiles of the *GATA6* (68.4%), *CD81* (46.1%), *DR4* (41.3%), and *CASP8* genes were confirmed by GBM patient data [69]. Verhaak et al. [13], proposed the GBM subtype classification by evaluating transcriptome profiles with a system-based approach: Proneural (high *PDGFRA* gene expression and frequent IDH1 mutation), neural (defects on *SYT1*, *SLC12A5*, *GABRA1,* and *NEFL*), classical (chr.7 amplification, chr.10 loss, *RB* inactivation, and *NES* overexpression), and mesenchymal (*P53*, *PTEN*, and *NF1*, overexpression in NF-κB pathway). Another study suggests the combined expression of *STAT3* and *C/EBPB*, which is associated with the mesenchymal state of GBM, as a prognostic biomarker for tumor aggressiveness [70].

### 3.5. Examples of Systems Biology Studies in Other Cancers

#### 3.5.1. Kidney Cancer

Renal cell carcinoma (RCC) is also a cancer type with highly individual heterogeneity, and 70% of RCC are clear cell renal carcinoma (ccRCC) [71]. Due to the high heterogeneity, many studies have applied systems biology approaches to stratify ccRCC into different subtypes, and then explored the oncogenic mechanism based on the distinct multi-omics characteristics or identified drug targets or effective drugs for each subtype. For example, Brannon et al. used unsupervised consensus clustering to classify the ccRCC patients into two subtypes (ccA and ccB) based on the gene expression microarray data [72]. The patients of ccA and ccB subtypes have different mRNA expression patterns and prognostic survival outcomes. Another well-known TCGA classification system stratified the ccRCC into four subtypes (m1-4) by the unsupervised clustering based on the mRNA-seq data [73]. These subtypes have different frequencies of genetic mutation and levels of DNA promoter methylation based on an integrated multi-omics analysis. Recently, Li et al. identified three subtypes of ccRCC with different prognoses, and identified a set of biomarkers that could robustly stratify the patients into different subtypes using the relative expression orders of biomarker genes [74].

Furthermore, they identified a shared drug target, SOAT1, by GEMs analysis and repositioned it as an FDA-approved drug, mitotane, to treat ccRCC. In terms of drug repositioning in ccRCC, a widely used method selects a drug that has a reversal perturbation on the gene expression of tumors compared to normal tissues as the therapeutic drug [75,76,77]. ConnectivityMap [78] is one of the most well-known drug perturbed gene expression databases, commonly used for drug repositioning studies. Various studies have focused on molecular subtype classification, biomarker, drug target identification, drug development/repositioning using different systems biology methods in kidney cancer. However, the considerations for promoting these biomarkers, which are used for clinical practice, repurposed drugs involved in drug combination, and alternative doses, must be further explored.

#### 3.5.2. HCC

Hepatocellular carcinoma (HCC), the primary form of liver cancer, has a very high mortality rate, partly due to its high heterogeneity. Therefore, it is one of the primary causes of cancer deaths globally. This makes it challenging to identify appropriate and effective therapeutic targets for dealing with HCC.

For this reason, systems biology approaches that can capture high tumor heterogeneity have been used in many studies to identify effective therapeutic targets [79]. For example, in comprehensive research, analysis of RNA expression, miRNA, protein expression, and DNA methylation derived from HCC patients revealed some highly mutated genes, such as *LZTR1*, *EEF1A1*, *SF3B1*, and *SMARCA4*. In the same study, by investigating integrative molecular subtyping with unsupervised clustering methods, three subtypes of HCC were identified, one of which was associated with a poor prognosis of HCC. In addition, the same study proposed potential therapeutic targets for which the inhibitor is available, including WNT signaling agents, such as TERT, MDM4, VEGFA, IDH1, MCL1, MET, as well as immune checkpoint proteins, such as CTLA-4, PD-1, and PD-L1 [80].

Cellular network analysis approaches are used to understand the biological aspect of heterogeneity in cancer, and determine potential therapeutic targets. Nevertheless, the functionality of the biological network may not be prioritized for ranking candidate targets, whilst avoiding toxicity to non-cancerous tissues. In this context, a novel systems biology approach has been adopted to overcome these problems by integrating gene expression data into GEMs [79]. In this study, network topology was used to rank therapeutic targets. Eventually, several potential targets were identified to inhibit cell growth, such as 74 different anticancer metabolites and the other three gene targets, PRKACA, PGS1, and CRLS1. Finally, the predicted anticancer metabolites were searched in the literature and found compatible with current FDA-approved cancer drugs. In addition, those three genes, PRKACA, PGS1, and CRLS, were experimentally confirmed with liver cancer cell lines HepG2 and Hep3B. These systems biology approaches demonstrate that they successfully identify therapeutic targets for the effective treatment of cancer [79].

#### 3.5.3. Drug Repositioning Applications in GBM

In recent years, the anticancer effects of repurposed drugs have gained significant interest due to the lack of directed treatments for life-threatening cancers [8,74,81]. Drug repurposing applications combine diverse approaches by complementing experimental and computational methodologies to discover targets in the fight against various cancers, including GBM [82]. In addition to its cost-effectiveness in drug development, repurposed molecules can readily be involved in clinical phase studies or applied to expanded access programs, particularly for patients with no alternative therapies. Moreover, drug repurposing methodologies provide a more comprehensive data source for exploring additional insights into the metabolic reprogramming of cancer, along with the drugs with originally unnoticed anticancer features that can expand in order to reveal the complex characteristics of tumor biology [83]. On the other hand, drug repurposing unveils previously unknown biological networks, developing them into distinct target biomolecules, despite the possibility that the identified targets may not be carried out in clinical studies [84].

Computational methods for drug repurposing can be widely classified as signature-based, network-based, chemical structure-based, and genome-wide association studies. Briefly, signature-based approaches compare the features of a molecule against another drug or disease using various omics or clinical phenotypes [85]. Similarly, in network-based drug repurposing, the drug and target/drug/disease interaction networks are constructed using the data according to gene expression profiles, protein-protein interactions or disease associations [86]. Another helpful drug repositioning method is estimating one molecule’s relative orientation and position at the binding site of another molecule by molecular docking methods. Finally, GWAS aims to identify differences in genetic material and helps in understanding the physiology of diseases accordingly [87].

Characterization of the cells at molecular levels, and detection of the relationship between pathways and numerous drug groups, provide an initial point for a successful precision medicine. In this manner, Johansson et al. analyzed the pharmacogenomic profile of patient-derived GBM cell cultures with 1544 drugs and stated that the response to proteasome inhibitors is correlated with *TP53* and *CDKN2A/B* variations [88]. In parallel, virtual screening of mutations in critical proteins (e.g., kinases) proposes the emergence of a robust strategy for candidate molecule identification from the available therapeutics targeting GBM. For instance, Bonnet et al. used a computational prediction algorithm to unveil molecular targets for mebendazole, an anthelminthic drug and found that 12 proteins significantly upregulated at GBM. Furthermore, molecular modeling showed that mebendazole was able to bind to MAPK14 [89]. Similarly, a molecular docking screening accompanied by cell-based validation to identify possible compounds against hnRNP, indicated that riluzole, an FDA-approved drug for amyotrophic lateral sclerosis, may be reutilized to treat GBM [90]. These studies support the concept that an anticancer molecule targeting kinase might be useful in combating the GBM. In summary, there are viable alternatives in the system-level that analyze the drug-target associations, in order to explore candidate proteins for drug repurposing.

## 4. Future Perspectives

Systems biology approaches are powerful tools to understand the flow of information in living systems. A wide variety of tools and methods could be used to gain meaningful insights into condition-specific cellular functioning. The analysis and integration of omics datasets provide a holistic understanding of biological processes and diseases, facilitating many studies. However, this brings some challenges in statistical analysis, data processing, and in combining various data. Moreover, due to the heterogeneity of individual omics data, large datasets, and the diversity of computational tools, the lack of studies that prioritize powerful tools and methodologies make systems-based multi-omics data integration analysis a confusing task.

Since omics data are produced using various platforms, the data storage and format type diverse significantly. Most of the systems biology analysis tools need specific data formats. Consequently, individual data must be pre-processed with specific data filtering, normalization, and quality control approaches. However, it is challenging to decide on an appropriate pre-processing approach due to the lack of universal standards, since in omics, integration tools that design for the same purpose could produce different results. In this context, developing new systems biology methods and tools or minimizing their differences in an inclusive platform could enable the efficient handling of large datasets. The primary step in the systems-based analysis is the selection of a suitable method to solve the biological question of interest. In this regard, researchers recently conducted comparative studies that comprehensively evaluate systems biology tools in order to select the most appropriate approach and methodology. More comprehensive comparative studies are essential for pioneering the scientific society in order to understand the wide range of tools. Another critical factor in the interpretation of omics data is clinical information. Therefore, robust methods could be developed for the integration of non-omics variables, such as epidemiological, demographic, and clinical metadata in order to understand the biological questions.

## Figures and Tables

**Table 1:** Omics sources for GBM.

Resources	Omics Layer	Notes	Reference
**Projects**			
TCGA	Genomics, Proteomics, Transcriptomics, Epigenomics		http://cancergenome.nih.gov/, accessed on 29 October 2021
CGP	Genomics, Proteomics, Transcriptomics, Epigenomics		https://www.sanger.ac.uk/, accessed on 25 October 2021
ICGC	Genomics, Transcriptomics, Epigenomics		https://daco.icgc.org/, accessed on 23 October 2021
CPTAC	Proteomics, Genomics		https://proteomics.cancer.gov/, accessed on 19 October 2021
**Databases**			
GEO	Genomics, Transcriptomic		https://www.ncbi.nlm.nih.gov/geo/, accessed on 19 October 2021
Expression Atlas	Genomics, Proteomics, Transcriptomics, Epigenomics, Interactomics		https://www.ebi.ac.uk/gxa/home, accessed on 29 October 2021
ArrayExpress	Genomics, Proteomics, Transcriptomics, Epigenomics, Interactomics		https://www.ebi.ac.uk/arrayexpress/, accessed on 23 October 2021
Human Protein Atlas	Proteomics, Transcriptomics		https://www.proteinatlas.org/, accessed on 25 October 2021
DDBJ	Genomics, Transcriptomics		https://www.ddbj.nig.ac.jp/, accessed on 24 October 2021
ENCODE	Genomics, Transcriptomics, Epigenomics		https://www.encodeproject.org/, accessed on 19 October 2021
StarBase	Interactomics	Pathway browser, Analysis tools	http://starbase.sysu.edu.cn/, accessed on 29 October 2021
BioGrid	Interactomics	Biological interaction, PPI	https://thebiogrid.org/, accessed on 22 October 2021
Reactome	Genomics, Proteomics, Transcriptomics, Interactomics	Reactions, Pathway browser, Analysis tools, Visualization	https://reactome.org/, accessed on 29 October 2021
KEGG	Proteomics, Transcriptomics, Proteomics, Interactomics	Reactions, Pathway browser, Analysis tools, Visualization	https://www.genome.jp/kegg/, accessed on 29 October 2021
STRING	Interactomics	Pathway browser, Analysis tools, Visualization	https://string-db.org/, accessed on 15 October 2021
HMDB	metabolomics	Pathway browser, Analysis tools	https://hmdb.ca/, accessed on 13 October 2021
GeneBank	Genomics	Analysis tools	https://www.ncbi.nlm.nih.gov/genbank/, accessed on 21 October 2021
Ensembl	Genomics	Genome browser, comparative genomics, Analysis tools	https://www.ensembl.org/, accessed on 21 October 2021
PRIDE	Proteomics	Analysis tools	https://www.ebi.ac.uk/pride/, accessed on 24 October 2021
Lipid Maps	Lipidomics	Analysis tools, Structure drawing	https://www.lipidmaps.org/, accessed on 29 October 2021
UniProt	Proteomics	Analysis tools	https://www.uniprot.org/, accessed on 29 October 2021
ChEBI	Metabolomics	Small chemical compounds	https://www.ebi.ac.uk/chebi/, accessed on 29 October 2021
MetaboLights	Metabolomics	Metabolomics repository	https://www.ebi.ac.uk/metabolights/, accessed on 29 October 2021
JASPAR	Interactomics	TF binding, Analysis tools	http://jaspar.genereg.net/, accessed on 25 October 2021
geneXplain	Interactomics	Analysis tools, TF binding	https://genexplain.com/, accessed on 22 October 2021
HPRD	Proteomics, Interactomics	Pathway browser, Analysis tools, PPI	http://www.hprd.org/, accessed on 18 October 2021
miRTarBase	Interactomics	miRNA-target interactions	http://miRTarBase.cuhk.edu.cn/, accessed on 12 October 2021
GWAS Catalog	Genomics	Genetic variant	https://www.ebi.ac.uk/gwas/, accessed on 19 October 2021
dbGAP	Genomics, Epigenomics	Genotypes and Phenotypes, Analysis tools	https://www.ncbi.nlm.nih.gov/gap/, accessed on 19 October 2021
dbSNP	Genomics	SNP genotyping	https://www.ncbi.nlm.nih.gov/snp/, accessed on 19 October 2021
**Tools**			
3Omics	Transcriptomics, Proteomics, Metabolomics	Pathway enrichment, correlation and co-expression network, ID conversion	https://3omics.cmdm.tw/, accessed on 29 October 2021
BioCyc and MetaCyc	Genomics, Proteomics, Metabolomics	Pathway, Enzymes, Reactions, Analysis tools	https://biocyc.org/, accessed on 23 October 2021
Cell Illustrator 5.0	Genomics, Transcriptomics, Proteomics	Visualize biological pathways	http://www.cellillustrator.com/home, accessed on 29 October 2021
CellML	Genomics, Transcriptomics, Proteomics	Mathematical modeling, XML markup language	https://www.cellml.org/, accessed on 21 October 2021
COBRA	Genomics, Transcriptomics, Proteomics	Constraint-based modeling, MATLAB	https://opencobra.github.io/cobratoolbox/stable/, accessed on 22 October 2021
RAVEN 2.0	Genomics, Proteomics	Genome-scale metabolic modeling, MATLAB	https://github.com/SysBioChalmers/RAVEN, accessed on 22 October 2021
Cytoscape	Genomics, Transcriptomics, Proteomics, Fluxomics	Visualizing and integrating pathways	https://cytoscape.org/, accessed on 14 October 2021
E-Cell	Genomics, Transcriptomics, Proteomics	Modeling, simulation, and analysis	https://www.e-cell.org/, accessed on 12 October 2021
Escher	Genomics, Proteomics, Metabolomics	Visualization of metabolic pathways	https://escher.github.io/#/, accessed on 19 October 2021
Gaggle	Genomics, Transcriptomics, Proteomics, Fluxomics	Integration of diverse database	https://isbscience.org/, accessed on 19 October 2021
IMPaLA	Transcriptomics, Proteomics, Metabolomics	Pathway analysis	http://impala.molgen.mpg.de/, accessed on 19 October 2021
Ingenuity Pathway Analysis	Transcriptomics, Proteomics, Metabolomics	Pathway analysis, commercial	https://digitalinsights.qiagen.com/products-overview/discovery-insights-portfolio/analysis-and-visualization/qiagen-ipa/, accessed on 25 October 2021
MarVis-Pathway	Transcriptomics, Metabolomics	Pathway browser, Visualization	http://marvis.gobics.de/, accessed on 25 October 2021
MassTrix	Metabolomics, Proteomics	Mapping, Analysis	http://masstrix.org/, accessed on 23 October 2021
MetaboAnalyst	Genomics, Transcriptomics, Proteomics, Metabolomics	Integrative Analysis	http://www.metaboanalyst.ca/, accessed on 29 October 2021
MetaboLights	Metabolomics	Database	http://www.ebi.ac.uk/metabolights/, accessed on 29 October 2021
MetScape 3	Transcriptomics, Metabolomics	Visualization, interpretation	http://metscape.ncibi.org/, accessed on 29 October 2021
mixOmics	Transcriptomics, Proteomics, Metabolomics	Integration and exploration of datasets	http://mixomics.org/, accessed on 29 October 2021
OmicsPLS	Transcriptomics, Proteomics, Metabolomics	Data integration, R	https://github.com/cran/OmicsPLS, accessed on 29 October 2021
Omickriging	Transcriptomics, Proteomics, Metabolomics, Fluxomics	Omics integration tools, R	https://cran.r-project.org/web/packages/OmicKriging/index.html, accessed on 21 October 2021
Omix visualization tool	Transcriptomics, Proteomics, Metabolomics, Fluxomics	Visualization and modeling, commercial	https://www.omix-visualization.com/#sthash.ScUNDhbD.dpbs, accessed on 21 October 2021
PaintOmics 3	Transcriptomics, Metabolomics	Integrative visualization	http://www.paintomics.org/, accessed on 29 October 2021
PathVisio 3	Transcriptomics, Proteomics, Metabolomics	Pathway creation and curation	https://pathvisio.github.io/, accessed on 21 October 2021
SimCell	Genomics, Proteomics, Transcriptomics, Metabolomics	Cell simulation	http://wishart.biology.ualberta.ca/SimCell/, accessed on 29 October 2021
VANTED	Transcriptomics, Proteomics, Metabolomics	Mapping, Processing, Analysis, Visualization	https://www.cls.uni-konstanz.de/software/vanted/, accessed on 24 October 2021
**Omics data integration methods for GEMs**			
tINIT	Transcriptomics, Proteomics	Task-driven model reconstruction algorithm	[34]
FASTCORE	Transcriptomics	Context specific metabolic modeling	[35]
E-Flux2	Transcriptomics	Infers fluxes from transcriptomic data	[36]
SPOT	Transcriptomics	Correlation between fluxes and enzymatic transcript	[36]
PROM	Transcriptomics	The probability of a gene being on-off in the inactivation of a TF	[37]
MADE	Transcriptomics, Proteomics	The algorithm uses DEG genes or proteins to generate a GEM	[38]
GIM3E	Transcriptomics, Proteomics, Metabolomics	An algorithm creates a condition-specific metabolic network based on the objective function, transcriptome, and metabolome	[39]

**Table 2:** Current generic metabolic models.

Current Generic GEMs	Reaction Number	Metabolite Number	Gene Number	References
HMR1	8100	6000	3668	[57]
HMR2	8181	6006	3765	[62]
Human 1	13,082	8378	3625	[63]
Recon 1	3744	2766	1905	[64]
Recon 2.2	7440	5063	2140	[65]
Recon 3D	13,543	4140	3288	[66]
iNL403	1070	987	403	[67]
iMS570	630	524	570	[61]
iHsa	8264	5620	2315	[68]
